# Reconstruction of Heel Soft Tissue Defects: An Algorithm Based on Our Experience

**DOI:** 10.29252/wjps.10.3.63

**Published:** 2021-09

**Authors:** Deepak Krishna, Gaurav Chaturvedi, Manal M Khan, Ved Prakash Rao Cheruvu, Michael Laitonjam, Reena Minz

**Affiliations:** 1 Department of Burns and Plastic Surgery, All India Institute of Medical Sciences, Bhopal, India

**Keywords:** Heel reconstruction, Extended reverse sural artery flap, Calcaneal osteomyelitis, Heel defect, Medial plantar artery flap

## Abstract

**BACKGROUND:**

Sensory recovery and durability of the flap is the primary goal of heel soft tissue reconstruction. From the different options, the choice of the flap depends on the size of the defect, its location, and the availability of the donor area.

**METHODS:**

In this retrospective study, 40 patients having heel defects were included from Jan 2016 to Dec 2018 in which different flaps were used for the reconstruction. The outcome was evaluated in terms of flap survival, recovery of sensation, the durability of coverage, and functional denouement. We also analysed the outcome between neuropathic and non-neuropathic ulcers of the heel.

**RESULTS:**

Out of 40 patients’ medial plantar artery islanded flap was performed in eight cases, extended reverse sural flap in 16 cases, islanded reverse sural flap in six cases, local flaps in six cases, cross-leg flap in two cases, and free Latissimus Dorsi muscle flaps with Skin Graft cover in two cases. The patients were observed for a mean follow-up time of 15 months (12-20 months). Only two flaps showed marginal necrosis as an immediate complication. The majority of the flaps were tenacious in the follow-up period except for the six flaps that developed delayed ulceration. Return of protective sensation (P=0.006) and mean American Orthopaedic Foot and Ankle Society subjective score (P=0.025) was significantly higher in the non-neuropathic ulcer group.

**CONCLUSION:**

Locoregional flaps can cover most of the heel defects with a satisfactory outcome. The functional outcome was lower in the reconstructed neuropathic heel ulcer group.

## INTRODUCTION

Reconstruction of the heel soft-tissue defect with sensate and durable tissue which can offer a productive life to the patient is still challenging. As the heel is a weight-bearing part of the body and subject to repetitive trauma, heel defects are quite common in the working Indian population.

The heel is a specialized tissue in the body having a thick epidermis and dermis, firmly anchored to the plantar aponeurosis by perpendicular fibrous Septa which prevents shearing force and fat loculi present between these vertically orientated septa provide a shock-absorbing function^1^^-^^3^.

Defects in this region can occur due to trauma, burns, tumor excision, or trophic ulcer. In the case of superadded infections, it may potentially lead to secondary amputation^4^^,^^5^. Trauma in the form of avulsion injury of the heel can lead to loss of total or isolated posterior heel, associated with exposed Achilles tendon, or calcaneal bone fracture^6^^-^^8^. On the other hand, Tumor, and Neurogenic ulcers commonly involve anterior weight wearing part of the heel. Neurogenic ulcers develop in patients with neuropathies like Hansen’s disease, diabetic neuropathies, syringomyelia, spina bifida, traumatic nerve injuries, etc. Because there is a loss of protective sensation in these patients and they do not relieve pressure intermittently over pressure points, it leads to skin breakdown and ulceration due to repetitive trauma^9^.

Reconstruction of the heel with similar tissue is challenging because of the limited availability of glabrous local tissue. Different options for reconstruction of the heel are skin graft, loco-regional flaps, cross-leg flap and different free flaps with each of them has their pros and cons^10^. 

This single-center study was conducted to evaluate the various flap options available to cover heel defects. We also entailed flap survivability, sensory recovery along with a comparison of functional outcomes between neuropathic and non-neuropathic heel ulcer groups. 

## MATERIALS AND METHODS

In this retrospective study, an analysis was performed using the medical records of 40 patients undergone soft tissue reconstruction of the heel at All India Institute of Medical Science, Bhopal, India from Jan 2016 to Dec 2018. 

The study was approved by the Institutional Review Board (Ethics code: IHEC-LOP/2020/IM0259) and informed consent has been obtained from all participants prior to enrolment. 

**Fig. 1:**
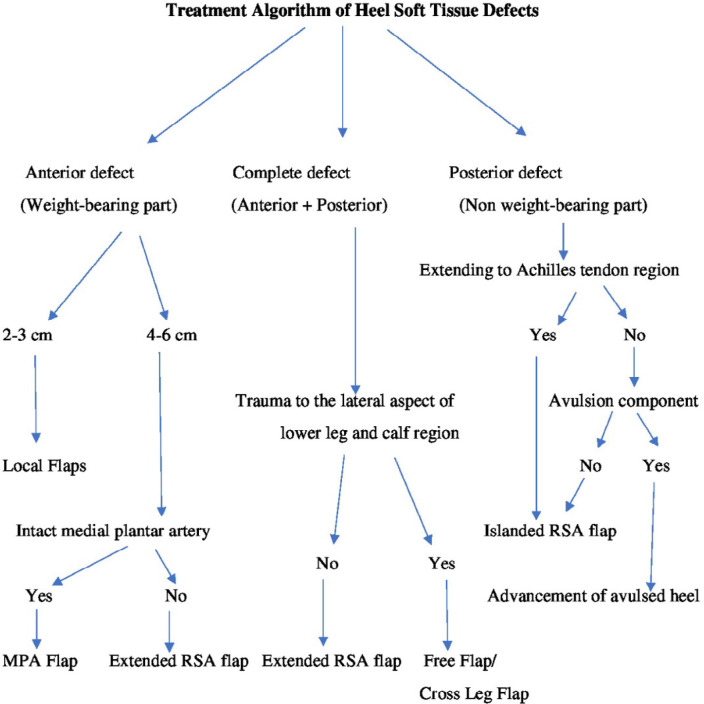
Treatment algorithm of heel soft tissue defects

Patient’s data including age, sex, concomitant medical illness, etiology, defect size, and the location was recorded. Wounds were prepared by debridement, daily dressing, and antibiotic coverage according to the wound swab culture report. The soft tissue defects of the heel were classified as anterior when located on the weight-bearing part of the heel, posterior when they were located on the non-weight-bearing part of the heel, and complete when involving the anterior as well as the posterior heel^7^^,^^11^. Based on the location of the heel defect, its size, and available options, various flaps were planned (Figure 1).

**Fig. 2:**
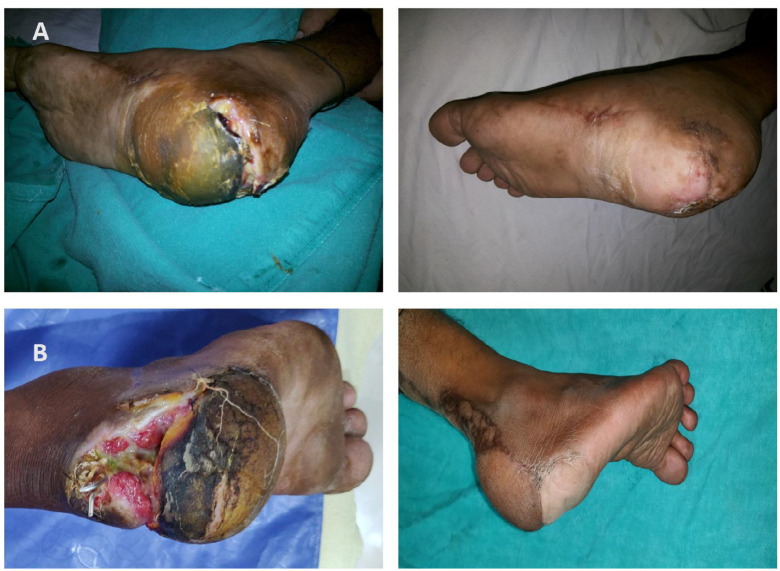
**A. **Pre- and Post-operative image of posterior heel defect with avulsion component managed with advancement of avulsed heel. **B.** Posterior heel defect extending up to Achilles tendon region covered with the islanded RSA flap

Local V-Y advancement and rotation flaps were utilized to cover small defects at the anterior heel region. Advancement of avulsed heel flaps was used to cover small defects at the posterior heel region. (Figure 2A).

**Fig. 3:**
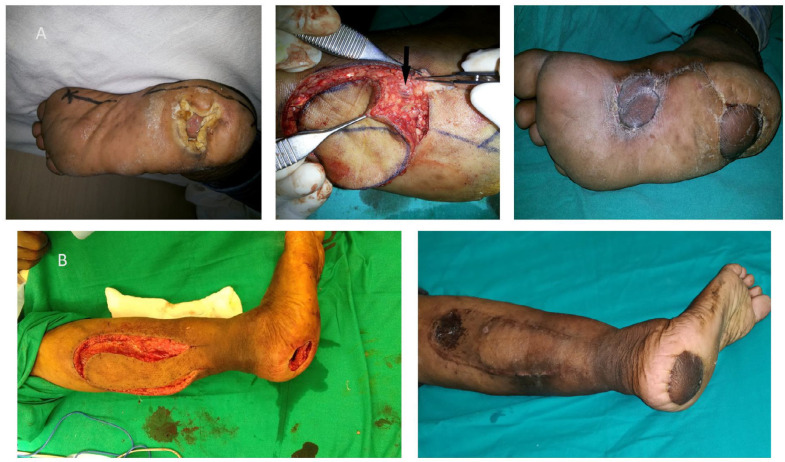
**A.** Neuropathic ulcer at right anterior heel (Left side), Black arrow indicate a subcutaneous vascular bundle of a medial plantar artery proximal to the flap (Middle), 2 months follow-up showing well-settled MPA flap and skin graft (Right side). **B.** Neuropathic ulcer of 4 cm x 4 cm size at the right anterior heel with elevated extended RSA flap (Left side), 3 months follow-up showing the well-settled flap (Right side)

Medial Plantar Artery islanded (MPA) flaps were harvested to cover moderate size defects at the anterior heel region (Figure 3A). Preoperatively Handheld Doppler was used to confirming the presence of the Medial Plantar artery. The MPA islanded flap was first described by Harrison and Morgan^12^ in which the distal end of the medial plantar artery was divided and elevated with the flap. Here all the Medial Plantar Artery flaps were raised based on the superficial subcutaneous branch of the medial plantar artery in spinal block anaesthesia under tourniquet control and loop magnification^13^. This approach prevents the sacrifice of the medial plantar artery and maintains its continuity. Cutaneous nerve included in flaps where the sensation of the sole was intact. After completion of flap dissection, an incision was made over intact skin between the donor and recipient area for the flap transposition, sutured after the flap inset. The donor site was covered by a split-thickness skin graft. Postoperative immobilization of the ankle joint was done with a dorsal splint till skin graft take-up. 

**Fig. 4:**
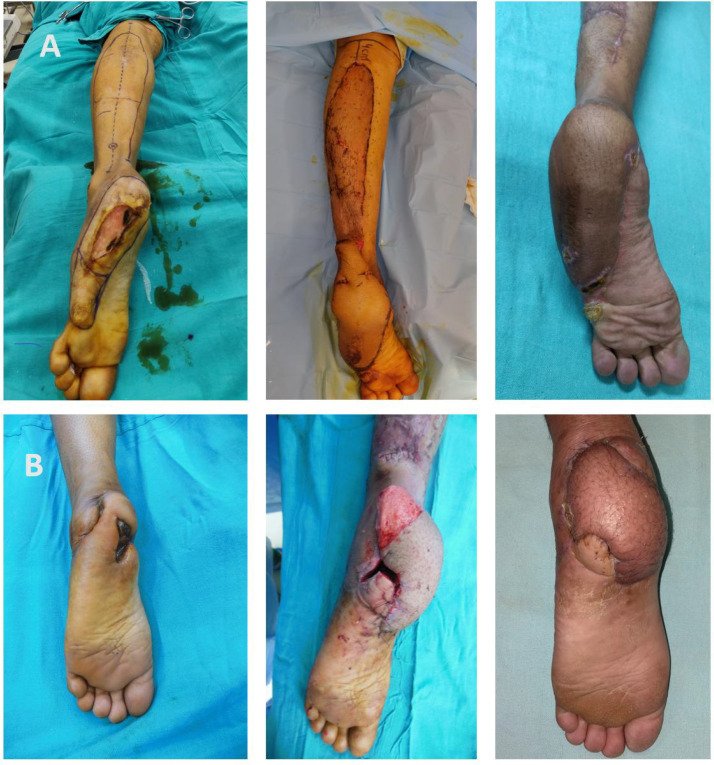
**A.** Posttraumatic complete heel defect at the lateral border of the left sole of size 8 cm x 4 cm size with surrounding scarring and marking extended RSA flap up to 4 cm proximal to the popliteal crease (Left side), Exteriorization of pedicle and reach of the flap up to the midfoot (Middle), 1-month follow-up showing 16 cm x 8 cm size flap (Right side). **B.** Post-traumatic defect at the left heel with the exposed calcaneum (Left side), In second after pedicle division de-epithelized part of the pedicle used for filling the bony cavity (Middle), 2 months follow-up showing complete healing (Right side)

Extended reverse sural artery (RSA) flaps were performed to cover complete heel defects (Figure 4A) as well as to cover defects at the anterior heel when the Medial plantar artery flap was not feasible (Figure 3B). Posterior heel defects without avulsion component and posterior defects extending up to the Achilles tendon region were covered by conventional islanded reverse sural artery flap with a pedicle width of four cm (Figure 2B). Extended RSA flap cover was accomplished in two stages with the exteriorization of the fasciocutaneous pedicle as described by ^14^ under spinal block anaesthesia with tourniquet control. In the first stage, the flap was marked over the upper third of the calf with a maximum upper limit of 4-5 cm distal to the popliteal crease. Flap elevated deep to fascia from proximal to distal including short saphenous vein and sural nerve. The pivot point of the flap was kept at five cm proximal to the tip of the lateral malleolus and the width of the fasciocutaneous pedicle was maintained around five cm between the posterior border of the fibula and midline of the Achilles tendon. The flap elevated and the defect was covered as an Interpositional flap. Donor sites were covered with a split-thickness skin graft. Immobilization of ankle joint done with a dorsal splint till flap division. The patient was kept in a contralateral decubitus position to avoid compression at the pedicle site. Flap division and final inset were done in the second stage after 3 wk, post flap delay, and pedicle transposed back to the donor site. 

Free Latissimus dorsi (LD) muscle flaps with skin graft cover were performed to cover complete heel defects with bare bone, where bulk is required to fill the defect. The Thoracodorsal artery was anastomosed with a posterior tibial artery in the end to side fashion while the vein was anastomosed with the posterior tibial vein in the end-to-end fashion. The donor site was closed primarily. 

Coverage of the complete heel defects by superiorly based medial fasciocutaneous cross-leg flaps was done as the last resort, where all the above-mentioned options were not possible.

In all patient’s weight-bearing was allowed 4 wk after complete suture removal. The objective assessment of outcome was performed in terms of flap survival, sensory recovery, the durability of coverage. The functional outcome comprising of limitations of activity, walking distance, and walking surfaces, and pain were assessed using the subjective component of the American Orthopaedic Foot and Ankle Society (AOFAS) hindfoot clinical ratings scale (Table 1)^10^^.^^15^^,^^16^. The sensation was objectively assessed by using the Semmes-Weinstein monofilament test at the flap site, with 3 different monofilament sizes (size 6.65=300 g for deep sensation, size 4.31=2 g for protective sensation, and size 3.61=0.4 g for normal touch sensation) ^10^^,^^17^. Soft tissue breakdown was noted clinically to assess the durability of the flap. 

***Statistical analysis***

The data were entered in an MS Excel spreadsheet and analysis was done using SPSS (ver. 21.0, Inc., Chicago, IL, USA). Categorical variables were presented in number and percentage (%), and continuous variables were presented as mean ± standard deviation and range. Variables were compared using an independent t-test between the two groups. A *P*-value ≤0.05 was considered statistically significant. 

## RESULTS

Analysis of 40 patients with heel defect and their details were described (Table 2). There were 35 males and 5 females, with a male to female ratio of 7:1. The mean age of the study participants was 39.37 yr; ranging from 21 to 55 yr. The most common cause of heel defect was traumatic injury (21; 52.5%) followed by neuropathic ulcer (12; 30%), burn injury (5; 12.5%), and malignancy (2; 5%). Osteomyelitis of calcaneum was found in 4 defects.

Different flaps performed for heel defects coverage and their outcome were assessed (Table 3). Out of the 16 extended RSA flaps, 12 were performed for complete and four for anterior defects. Out of six islanded RSA flaps, two were used for isolated posterior defects and four for posterior defects extending up to the Achilles tendon region. Out of six local flaps, four were done for anterior defects and two heel advancement flaps were utilized for posterior defects. Both free LD muscle and cross leg flap were utilized for complete defects. 

Most of the flaps survived well except one case of local rotation flap and another that of extended RSA flap in which marginal necrosis occurred. Tip necrosis of the rotation flap was managed with debridement followed by secondary suturing while marginal necrosis of the extended RSA flap was managed with secondary healing. Most flaps were healthy in the mean follow-up period of 15 months (12-20 months) except four cases of the extended sural flap and two cases of medial plantar artery flap in which ulceration developed at flap margin managed with regular dressings. All patients gained deep sensation at an average period of 5.8 months (3-8months), but only 26 patients had protective sensation with a mean of 9.6 months (8-14 months). Only two cases of medial plantar artery flaps, used after tumor excision, and two cases of the advancement of avulsed heel flaps used for posterior heel defects showed touch sensation at 10.75 months (11-12 months). 

Return of protective sensation and mean AOFAS subjective score was significantly higher in the non-neuropathic ulcer group (Table 4). 

## DISCUSSION

Reconstruction of Heel defect with durable tissue which can bear the load of daily activity and offers to bear of the normal shoe is the primary goal of surgery^7^. Based on the location of heel defects, it may be anterior, posterior, and complete. Isolated anterior heel defects at the weight-bearing part mostly occur as a consequence of neuropathies, tumor excision, and burn injury^7^^,^^9^. Longstanding anterior heel defects many times associated with osteomyelitis of calcaneum and foul-smelling discharge, which should be addressed during management. For the reconstruction of defects at the anterior weight-bearing part of the heel, the medial plantar artery, and local flaps are preferred because they have a thick epidermis and dermis which is similar to heel skin^4^^,^^7^^,^^10^. Use of local flaps like V-Y advancement, transposition, and rotation usually limited to cover small size anterior heel defects^9^^,^^18^^-^^20^. Medial plantar artery islanded flap best suited for moderate-sized anterior heel defects in terms of matching, durability, and cosmesis. If there is a trauma in the instep region or the course of the medial plantar artery, then other regional flaps like distally based fasciocutaneous flaps from the leg or free flaps can be used for reconstruction of the weight-bearing part of the heel. 

In our study, most of the anterior defects were neuropathic in origin and, the rest were post-tumor excision and related to burn injuries. Small-sized anterior heel defects were covered with local suprafascial flaps like rotation, V-Y advancement flaps. Whenever possible, moderate-sized defects were covered with MPA islanded flaps based on the superficial branch of the medial plantar artery. In situations where the medial plantar artery flap was not possible, we performed an extended RSA flap along with the exteriorization of the fasciocutaneous pedicle to reconstruct anterior heel defects. Although it is a two-stage procedure main advantage over a single-stage sural flap is it maintains the contour of the uninvolved posterior heel and avoids unnecessary scar over the Achilles tendon region which may sometimes become painful while walking and also cosmetically not appealing (Figure 3B)^14^^,^^21^.

Defects at the posterior non-weight bearing part of the heel which can occur due to prolonged immobilization or traumatic avulsion injury of the heel, it does not require coverage with thick glabrous plantar skin. Defects in this region are commonly associated with Calcaneum fracture or injury to the Achilles tendon region^7^^,^^8^. Skin grafting is indicated if the wound is superficial. In most of the studies Reverse sural artery and lateral calcaneal artery flaps are the commonly used procedure if the defect is deep with exposure of calcaneum or Achilles tendon^6^^-^^8^^,^^22^. We were able to close posterior heel defects due to avulsion injury by debridement and advancement of the avulsed heel flap. Posterior heel defects without avulsion component and posterior defects extending to the Achilles tendon region were covered by an islanded RSA flap. Posterior heel defects in this study were covered by conventional RSA flap because these defects were small in size as well as the distance between the defect and pivot point is not much, due to this skin paddle lies over the suprafascial course of the sural nerve and easily reach the site of the defect in a single stage. 

For the reconstruction of a complete heel defect which involves both weight and non-weight-bearing part of the heel, local fasciocutaneous flaps are not adequate in size to cover the entire defect. Therefore, other options like reverse sural artery flap, distally based fasciocutaneous flap, free fasciocutaneous or muscle flap, cross-leg flap, and combination of two loco-regional flaps have to take into consideration. Large defects at the heel usually treated by free flaps but require expertise and infrastructure for microsurgery. Conventional RSA flap has a limited role in the reconstruction of large and distal foot defects, because of its described territory and unpredictable outcome^23^^-^^25^. Different modification of reverse sural flap-like flap delay, skin extension over pedicle, venous supercharging of the flap, etc. have been described to minimize the risk of venous congestion, flap necrosis and, also to expand the territory of the flap^14^^,^^25^^-^^31^. These modifications make the sural flap a reliable alternative to free tissue transfer in coverage of large foot defects and in patients with comorbidities it may be the preferred option. 

We prefer extended RSA flap as a first choice to reconstruct complete heel defects because, it is an easy, safe, less time-consuming procedure and not requiring any additional microsurgical procedure. An Extended RSA flap can be done single stage by splitting the intervening area lying between the pivot point and defect, or in two stages by exteriorization of the pedicle over the intervening area. The inclusion of skin over the pedicle has many advantages, as it provides additional blood supply as well as protect the pedicle from traction and twisting^14^^,^^27^^,^^28^. We did not encounter any venous congestion and major flap necrosis with this technique, even when the flap reaching up to the midsole region (Figure 4 A). Although this technique requires second surgery for pedicle division, extra wound care at the pedicle site, and donor site morbidity at the calf region, but well accepted by the patients. In the two-stage flap technique, at the time of flap division and inset, the de-epithelized part of the pedicle can be utilized to fill the partial bony cavity in the case of calcaneal osteomyelitis (Figure 4 B). Other techniques described in the literature to fill the partial bony cavity with soft tissue after calcaneal debridement are musculocutaneous sural artery flap and combined muscle and skin flaps of the calf^32^^,^^33^. 

Our experience in free flaps is only with free LD muscle flap used in very large complete heel defects with bared bone. Cross leg flap should be discouraged because it gives a lot of discomfort to the patient, as it required long-standing immobilization in difficult positioning. We used cross-leg flap in the relative indication of heel reconstruction when other mentioned flaps option was not feasible.

The drawback of the distally based flaps from the leg is; they are nonsensate and skin texture not similar to planter skin but in the long term, adaptive changes occur and protective sensation returns in the flap^6^^,^^10^. The advantage of the sensate free flap is an early return to daily living activity without the need for protective shoes^34^; however, in the long run, patients with nonsensate free flap did not report difficulty in daily living activity compared with the sensate group. Santanelli et al^17^ achieved most of the desired outcomes after flap surgery for the foot defects, like contour, stability, sensation, walking ability in a 1year postoperative follow-up period. In our study also most of the nonsensate flaps showed a return of protective sensation, which was higher in the non-neuropathic ulcer group 22/28 (78.5%) compared to the neuropathic ulcer group 4/13 (33.3%). The reason behind the return of protective sensation in the neuropathic ulcer group might be due to small anterior neuropathic heel ulcers with isolated peripheral nerve injury and intact surrounding sensation.

 In all the patients there was no abnormality in the skeletal framework of the foot. In our study delayed ulceration was observed in 2/8 (25%) MPA flaps and 4/16 (25%) extended RSA flaps. Schwarz and Negrini^35^ reported delayed ulceration in 7/50 (14%) MPA flaps performed for heel reconstruction. Yucel et al^1^observed the recurrence of ulceration in 1 out of 20 cases in his study on soft tissue reconstruction of sole and heel defects with free tissue transfer. In a study on seven cases of heel pad reconstruction^10^, all the flaps remained well without ulcer formation in the follow-up period. J.-x. Gu et al^13^ did not notice any delayed ulceration in 11 cases of heel reconstruction with MPA flaps but no case had neuropathic etiology. Benito-Ruiz et al^7^ did not notice recurrent ulcers in MPA and reverse sural flaps done for heel soft tissue reconstruction with 1 to 2 years of follow-up. Other studies, that used reverse sural flap for heel defects did not mention the recurrence of ulceration in their follow-up period^6^^,^^25^^,^^26^^,^^28^^,^^36^. 

No relevant differences was found in the functional results between different flap types and free or pedicled techniques used for reconstruction of the weight-bearing sole^4^. In our study mean AOFAS subjective score was highest for islanded RSA flap used in posterior traumatic defects (mean score of 54.66 of 60 points), followed by local flaps performed for small anterior and posterior defects with intact surrounding sensation (51.33 of 60 points). We found a lower mean AOFAS subjective score of MPA flap (40.5 of 60 points) compared to other studies^10^ in which the score was 57.3 of 60 points. The reason behind, this low AOFAS score in MPA flap could be its use mostly in neuropathic ulcers. 

**Table 1:** Subjective component of AOFAS hindfoot clinical ratings scale (Maximum score 60)

**I Pain (maximum 40 points)**	
None	40
Mild, occasional	30
Moderate, daily	20
Severe, almost always present	0
**II Functional (maximum 20 points)**	
Activity limitations, support requirement	
No limitations, no support	10
No limitation of daily activities, limitation of recreational activities, no support	7
Limited daily and recreational activities, cane	4
Severe limitation of daily and recreational activities, walker, crutches, wheelchair, brace	0
Maximum distance walking, blocks (1block= 150meter)	
Greater than 6	5
4-6	4
1-3	2
Less than 1	0
Walking surfaces	
No difficulty on any surface	5
Some difficulty on uneven terrain, stairs, inclines, ladders	3
Severe difficulty on uneven terrain, stairs, inclines, ladders	0

**Table 2:** Demographic profile

**Characteristic**	**Number of patients**
**Sex**	
Male	35 (87.5)
Female	5 (12.5)
Male: Female ratio	7:1
**Comorbidities**	
Diabetes	6
Hypertension	4
Varicosity	2
Peripheral nerve injury	8
**Etiology of defect**	
Trauma	21 (52.5%)
Neuropathic	12 (30%)
Burn	
Thermal	3 (7.5%)
Contact	2 (5%)
Malignancy	2 (5%)
**Location of the defect**	
Anterior	16 (40%)
Posterior	8 (20%)
Complete	16 (40%)

**Table 3:** Outcome analysis of reconstructed heel with different flaps

Flap type	Cases (n)	Average size in cm	Complication (n)		Mean AOFAS Score and range (Maximum Mean Score 60)	Sensory recovery in number of cases (n)		
			Immediate (Marginal necrosis)	Delayed (Ulceration)		Deep	Protective	Touch
MPA	8	4×4	0	2	40.50 (44-60)	8	2	2
Extended RSA	16	9.5×8	1	4	40.12 (34-47)	16	10	0
Islanded RSA	6	6×5	0	0	54.66 (44-60)	6	6	0
Local	6	7×5.2	1	0	51.33 (44-60)	6	6	2
Cross leg	2	8×6.5	0	0	44	2	2	0
Free	2	15×14	0	0	42	2	0	0

**Table 4:** Outcome analysis between Neuropathic and non-neuropathic heel defect groups

Group	Delayed ulceration (n)	Return of protective sensation (n)	Mean AOFAS Score and range (Maximum Mean Score 60)
Neuropathic (n=12)	3 (25%)	4 (33.3%)	41 ± 10.7
Non-neuropathic (n=28)	3 (10.7%)	22 (78.5%)	47.5± 6.7
**(P** **≤0.05) is significant**	**0.250**	**0.006**	**0.025**

## CONCLUSION

For small defects, at the anterior or posterior heel region local rotation and advancement flaps are very useful without donor site morbidity. Medial plantar artery flap for moderate size anterior heel defects, extended reverse sural flap for complete heel defects, and islanded reverse sural artery flap for posterior heel defects can cover most of the Heel defect with a good durable cover and functional outcome. Return of protective sensation and functional outcome was significantly higher in the non-neuropathic ulcer group.

## FINANCIAL SUPPORT

Nil.

## CONFLICTS OF INTEREST

There are no conflicts of interest.
